# Expression of IL-20 in synovium and lesional skin of patients with psoriatic arthritis: differential response to alefacept treatment

**DOI:** 10.1186/ar4038

**Published:** 2012-09-24

**Authors:** Maria C Lebre, Christina L Jonckheere, Maarten C Kraan, Arno WR van Kuijk, Jan D Bos, Menno de Rie, Danielle M Gerlag, Paul P Tak

**Affiliations:** 1 Division of Clinical Immunology and Rheumatology, Academic Medical Center/University of Amsterdam, Amsterdam, The Netherlands; 2 Department of Experimental Immunology, Academic Medical Center/University of Amsterdam, Amsterdam, The Netherlands; 3 AstraZeneca, Mölndal, Sweden; 4 Department of Dermatology, Academic Medical Center/University of Amsterdam, Amsterdam, The Netherlands; 5 Department of Dermatology, Erasmus Medical Center, Rotterdam, The Netherlands; 6 Academic Medical Center/University of Amsterdam and GlaxoSmithKline, Stevenage, UK

## Abstract

**Introduction:**

Psoriatic arthritis (PsA) is an inflammatory joint disease associated with psoriasis. Alefacept (a lymphocyte function-associated antigen (LFA)-3 Ig fusion protein that binds to CD2 and functions as an antagonist to T-cell activation) has been shown to result in improvement in psoriasis but has limited effectiveness in PsA. Interleukin-20 (IL-20) is a key proinflammatory cytokine involved in the pathogenesis of psoriasis. The effects of alefacept treatment on IL-20 expression in the synovium of patients with psoriasis and PsA are currently unknown.

**Methods:**

Eleven patients with active PsA and chronic plaque psoriasis were treated with alefacept (7.5 mg per week for 12 weeks) in an open-label study. Skin biopsies were taken before and after 1 and 6 weeks, whereas synovial biopsies were obtained before and 4 and 12 weeks after treatment. Synovial biopsies from patients with rheumatoid arthritis (RA) (*n *= 10) were used as disease controls. Immunohistochemical analysis was performed to detect IL-20 expression, and stained synovial tissue sections were evaluated with digital image analysis. Double staining was performed with IL-20 and CD68 (macrophages), and conversely with CD55 (fibroblast-like synoviocytes, FLSs) to determine the phenotype of IL-20-positive cells in PsA synovium. IL-20 expression in skin sections (*n *= 6) was analyzed semiquantitatively.

**Results:**

IL-20 was abundantly expressed in both PsA and RA synovial tissues. In inflamed PsA synovium, CD68^+ ^macrophages and CD55^+ ^FLSs coexpressed IL-20, and its expression correlated with the numbers of FLSs. IL-20 expression in lesional skin of PsA patients decreased significantly (*P *= 0.04) 6 weeks after treatment and correlated positively with the Psoriasis Area and Severity Index (PASI). IL-20 expression in PsA synovium was not affected by alefacept.

**Conclusions:**

Conceivably, the relatively limited effectiveness of alefacept in PsA patients (compared with anti-tumor necrosis factor (TNF) therapy) might be explained in part by persistent FLS-derived IL-20 expression.

## Introduction

Psoriatic arthritis (PsA) is an inflammatory joint disease associated with psoriasis, characterized by a heterogeneous clinical presentation including spinal involvement, peripheral synovitis, and enthesitis. Cellular infiltration plays an important role in the induction of inflammation in joint tissues, as well as in psoriatic skin [1].

Alefacept is a lymphocyte function-associated antigen (LFA)-3 immunoglobulin (Ig) fusion protein that binds to CD2 and functions as an antagonist to T-cell activation. Alefacept was the first of the biologic agents to be approved in the United States for the treatment of adult patients with moderate-to-severe chronic plaque psoriasis who are candidates for systemic therapy or phototherapy. This fully human fusion protein inhibits activation of memory T cells (CD45RO^+^), a subpopulation of lymphocytes that plays a critical role in the pathogenesis of psoriasis [2]. This therapy has been shown to be effective in patients with psoriasis [3], and it also has some efficacy in PsA [4,5] compared with anti-TNF therapy. Clinical response, as determined by the American College of Rheumatology 20 (ACR20) response at week 24, was achieved by a significantly greater proportion of patients receiving alefacept plus methotrexate (54%) compared with those receiving placebo plus methotrexate (23%), but proportions of patients achieving ACR50 and ACR70 responses at week 24 were not significantly different in a randomized, double-blind, placebo-controlled study in 185 PsA patients [5].

Interleukin-20 (IL-20) belongs to the IL-10 cytokine family. IL-20 receptor (IL-20R) and IL-20 are expressed in several normal tissue types, including the lungs, skin, and kidney [6,7]. Moreover, IL-20 has been implicated to play an important role in several autoimmune diseases that include rheumatoid arthritis (RA), lupus nephritis, and Crohn disease (reviewed in [8]). Recent reports have shown that IL-20 functions as a proinflammatory cytokine in several inflammatory diseases, of which psoriasis has been the most extensively studied [9,10]. *IL-20 *gene and protein expression was elevated in lesional psoriatic skin compared with normal and nonlesional skin [11-13]. Interestingly, its expression in PsA has as yet not been investigated. IL-20 is expressed mainly by activated monocytes [14]. Other sources of IL-20 are keratinocytes (KCs) [14,15], maturing dendritic cells (DCs) [16], synovial fibroblasts [17], endothelial cells [18,19], and renal mesangial cells [20]. IL-20 signals through two alternative receptor complexes: type I, which is composed of IL-20R1/IL-20R2 chains, and type II, which consists of an IL-22R/IL-20R2 heterodimer [21]. IL-20R1, IL-20R2, and IL-22R chains are coexpressed at high levels in the skin [7,22]. In addition, KCs [23,24], rheumatoid synovium (fibroblasts and synovial fluid cells) [17,24,25], and endothelial cells [17] express IL-20R chains. The interaction between IL-20 and its receptors leads to various biologic effects that include hyperproliferation of KCs [7], production of inflammatory cytokines and chemokines by synovial fibroblasts [17] and KCs [7], neutrophil chemotaxis, and angiogenesis [17]. Recently, a role for IL-20 in osteoclast differentiation was reported [26].

Decreased expression of IL-20 in the skin has been observed after effective treatment with calcipitriol, cyclosporine [22], and infliximab [27]. Of interest, reduced IL-20 expression has also been demonstrated in the skin of patients after alefacept treatment [13], but only in those who responded clinically (12 responders versus eight nonresponders). Thus, these initial data suggest that IL-20 might serve as a biomarker associated with efficacy in patients with psoriasis. Here, we extend the findings for the skin of patients with psoriasis and demonstrate for the first time the effects of alefacept treatment on IL-20 expression in the synovium of patients with PsA. Moreover, in view of these data, it is clear that IL-20 is a key proinflammatory cytokine for both skin and synovium. The unique use of paired skin and synovium from patients with PsA (with active psoriasis) allowed us to address the question whether alefacept treatment leads to a specific tissue-response mechanism.

## Methods

### Patients and tissue samples

Skin and synovial biopsy specimens were obtained from patients with chronic plaque psoriasis and PsA (*n *= 11 synovial biopsies; *n *= 6 skin biopsies) in a prospective open-label clinical trial, as previously described [4]. Clinical characteristics have been previously reported [4]. Patients received a weekly dose of alefacept (LFA-3TIP, anti-CD2; Amevive, Biogen, San Diego, CA, USA; 7.5 mg, intravenously) for 12 weeks. In brief, skin biopsies were taken before and after 1 and after 6 weeks, whereas synovial biopsies were taken before and 4 and 12 weeks after treatment. Synovial biopsy specimens from active RA patients (Table 1; *n *= 10) served as disease control group for IL-20 expression at baseline. Patient's disease activity score (DAS28), visual analogue scale (VAS) for global disease activity (scale, 0 to 100 mm), swollen-joint count (SJC), tender-joint count (TJC), erythrocyte sedimentation rate (ESR), and serum levels of C-reactive protein (CRP) were used to evaluate disease activity. The study was conducted according to International Conference of Harmonization (ICH)/Declaration of Helsinki, approved by the Medical Ethics Committee of Academic Medical Center (AMC)/University of Amsterdam, and all patients gave written informed consent. Psoriasis area and severity index (PASI) was assessed as previously described [28].

**Table 1:** Demographic and clinical characteristics of RA patients at baseline

	RA patients
Sex, female/male (*n*)	8/2 (10)

Age in years, mean (range)	55.5 (44-64)

DAS28	4.9 (2.9-6.5)

SJC, mean (range)	11.7 (6-18)

TJC, mean (range)	9.8 (1-16)

VAS, mean (range)	22 (10-32)

CRP (mg/l), mean (range)	21 (3-52)

ESR mm/h, mean (range)	23.9 (3-74)

### Synovial and skin biopsy immunohistochemical staining

All samples were, immediately after collection, embedded *en bloc *in Tissue Tec OCT (Miles, Elkhart, IN, USA) and subsequently snap frozen. The frozen blocks were stored in liquid nitrogen until processed. Shortly before staining, 5-μm sections were cut and mounted on glass slides (Star Frost; Knittelgläser, Braunschweig, Germany). The glass slides were sealed and stored at -80ºC until immunohistochemical analysis was performed.

In brief, after fixation with acetone, endogenous peroxidase activity was inhibited by using 0.1% sodium azide and 0.3% hydrogen peroxide in phosphate-buffered saline (PBS). The primary antibody against human IL-20 (Zymogenetics, Seattle, WA, USA) was incubated overnight at 4ºC followed by secondary antibody affinity-purified horseradish peroxidase (HRP)-conjugated goat anti-mouse (Dako Cytomation, Glostrup, Denmark) for 30 minutes, followed by subsequent incubation with biotinylated tyramide (Perkin Elmer, Boston, MA, USA) for 30 minutes and HRP-conjugated streptavidin for 30 minutes. HRP activity was detected by using hydrogen peroxide as substrate and amino ethylcarbazole (AEC; SK-4200; Vector Laboratories, Burlingame, CA, USA) as dye. With this procedure, IL-20-positive cells stained red. Slides were counterstained with Mayer hematoxylin (Merck, Darmstadt, Germany) and, after washing with distilled water, mounted in Kayser glycerol gelatin (Merck).

To identify IL-20-expressing cells in PsA synovial tissue, double staining was performed by using anti-IL-20 antibody together with FITC-labeled anti-CD55 (M2192; Sanquin Reagents, Amsterdam, The Netherlands) or FITC-labeled anti-CD68 (clone KP1; Dakocytomation). Fixation and blocking endogenous peroxidase activity was performed as stated earlier. The primary antibody against human IL-20 (Zymogenetics) was incubated overnight at 4ºC followed by secondary antibody affinity-purified horseradish peroxidase (HRP)-conjugated goat anti-mouse (Dako Cytomation) for 30 minutes, and subsequent incubation with biotinylated tyramide (Perkin Elmer) for 30 minutes and HRP-conjugated streptavidin for 30 minutes. After a blocking step of 15 minutes with 10% mouse serum (Sanquin Reagents), FITC-labeled anti-CD55 or FITC-labeled anti-CD68 was added to the sections and incubated for 1 hour at room temperature. Then the sections were incubated for 30 minutes with rabbit anti-FITC followed by alkaline phosphatase-conjugated swine anti-rabbit secondary antibodies (both from Dako Cytomation). HRP activity was detected as stated earlier. Alkaline phosphatase activity was detected by using the Alkaline Phosphatase Substrate III kit (SK-5300; Vector Laboratories). With this procedure, IL-20-positive cells stained red, CD55- or CD68-positive cells stained blue, and double-positive cells stained purple.

### Quantification of IL-20-expressing cells in skin and synovium

Skin biopsy specimens were scored semiquantitatively for IL-20 expression on a 5-point scale by two independent observers who were not aware of the clinical data. The scoring methods were described previously [29]. In brief, a score of 0 represented minimal staining, whereas a score of 4 represented widespread expression of IL-20 in this case. Individual readings were identical or differed by only 1 point. Minor differences between the observers were resolved by mutual agreement.

Quantification of IL-20-expressing cells in PsA and RA synovial tissues was performed with computer-assisted image analysis, as previously described [30]. The same holds true for CD68 and CD55 quantification. In brief, after immunohistochemical staining, all coded sections (one section per patient per time point) were randomly analyzed (18 high-power fields from different parts of the section were analyzed; for example, the mean of the 18 high-power fields was calculated). The images of the high-power fields were analyzed by using the Qwin analysis system (Leica, Cambridge, UK). Positive staining of CD68 and CD55 was expressed as positive cells/mm^2^. Positive staining for IL-20 was expressed as integrated optical density (IOD/mm^2^).

### Statistical analysis

SPSS version 15.0 for Windows (SPSS, Chicago, IL, USA) was used for statistical analysis. The Wilcoxon signed ranks test was used for comparison of nonparametric variables in related samples, and the Mann-Whitney *U *test was used for comparison of nonparametric variables in independent samples. Correlations between synovial IL-20 expression and disease activity score 28 (DAS28), synovial CD4, CD8, CD68, and CD55 were analyzed with Spearman rank correlation. Correlations between skin IL-20 and PASI were analyzed with Spearman rank correlation.

## Results

### Expression of IL-20 in PsA and RA synovia

**Figure 1:**
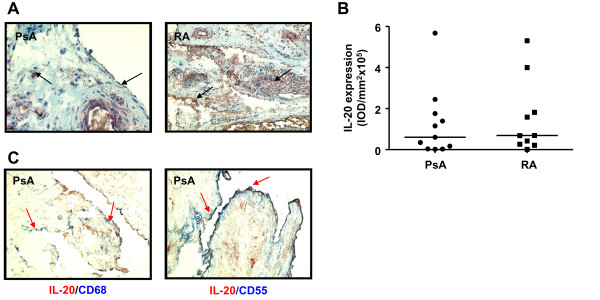
**IL-20 is expressed in PsA and RA synovial tissues**. **(A) **Representative immunohistochemical staining of baseline IL-20 expression in synovial tissue from PsA and RA patients. Arrows indicate expression of IL-20 in both lining and sublining. **(B) **Quantification of IL-20 expression in RA and PsA synovial tissues. Results are shown as median IOD (integrated optical density)/mm^2 ^× 10^5 ^of 10 patients with RA and 11 patients with PsA. **(C) **Double-immunohistochemistry stainings of IL-20 (red) and CD68^+ ^macrophages (blue) and CD55^+ ^FLS (blue) in PsA synovium. A representative double immunostaining of PsA synovium from one patient is shown. Arrows indicate double-positive cells. Original magnification, ×200. CRP, C-reactive protein; DAS28, disease activity score; ESR, erythrocyte sedimentation rate; SJC, swollen-joint count; TJC, tender-joint count; VAS, visual analogue scale (100 mm).

First we analyzed whether IL-20 expression in PsA and RA synovia differ from each other. IL-20 was expressed in the intimal lining layer, in the synovial sublining, and on endothelium in both PsA and RA patients (Figure 1A). Digital image analysis of synovial tissue demonstrated clear IL-20 expression with comparable overall scores in both patient groups (median Integrated Optical Density (IOD) (range)): PsA, 60,064 (1,588 to 567,696); RA, 68,554 (1,171 to 530,218) (Figure 1B). Double staining of inflamed PsA synovium showed that CD68^+ ^macrophages and CD55^+ ^FLS coexpress IL-20 (Figure 1C).

### Alefacept treatment does not affect IL-20 expression in PsA synovium

**Figure 2:**
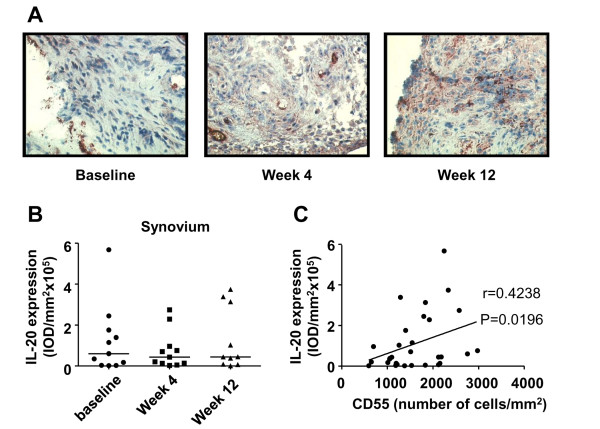
**Alefacept treatment does not affect IL-20 expression in PsA synovium**. **(A) **Representative immunohistochemical staining of IL-20 in the synovium of a PsA patient. **(B) **Quantification of IL-20 expression in PsA synovium. Results are shown as median IOD (integrated optical density)/mm^2 ^× 10^5 ^of 11 patients with PsA. **(C) **Correlation between the expression of IL-20 and the number of CD55^+ ^FLSs in PsA synovium. Original magnification, ×200. Each dot represents the mean of the 18 high-power fields per patient per time point. CRP, C-reactive protein; DAS28, disease activity score; ESR, erythrocyte sedimentation rate; SJC, swollen-joint count; TJC, tender-joint count; VAS, visual analogue scale (100 mm).

As previously described, alefacept treatment resulted in clinical improvement and in a reduction of CD4^+ ^T cells and CD68^+ ^macrophages in the synovial infiltrate [4]. Despite the clinical improvement and change in cellular infiltrate [4], no change was found in IL-20 expression in the synovium before and after treatment (Figure 2A and 2B). As CD68^+ ^macrophages and CD55^+ ^FLSs express IL-20, we investigated whether the levels of synovial IL-20 were associated with the numbers of these cells present in synovial tissue. Although no correlation was present between the levels of IL-20 and the numbers of both total CD68^+ ^and sublining CD68^+ ^macrophages (data not shown), the levels of IL-20 were positively correlated with the numbers of CD55^+ ^FLSs (*r *= 0.4238; *P *= 0.0196). In addition, the levels of synovial IL-20 expression were not correlated with disease-activity parameters: DAS28, C-reactive protein (CRP), and erythrocyte sedimentation rate (ESR) (data not shown).

### Decreased IL-20 expression in PsA skin lesions after alefacept treatment

**Figure 3:**
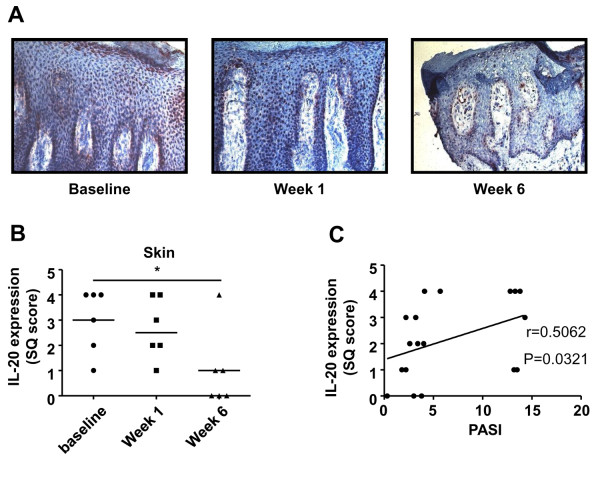
**Decreased IL-20 expression in PsA skin lesions after alefacept treatment**. **(A) **Representative immunohistochemical staining of IL-20 expression in the lesional skin of a PsA patient. **(B) **Quantification of IL-20 expression in PsA lesional skin. Results are shown as median of semiquantitative score (SQ) of six patients with PsA. **(C) **IL-20 expression in PsA lesional skin is correlated with disease activity (PASI). All time points are depicted (each dot represents one patient per time point). *Statistical significance, *P *= 0.04. Original magnification, ×200. CRP, C-reactive protein; DAS28, disease activity score; ESR, erythrocyte sedimentation rate; SJC, swollen-joint count; TJC, tender-joint count; VAS, visual analogue scale (100 mm).

In contrast to findings in the synovium, IL-20 expression in PsA lesional skin was significantly decreased (*P *= 0.04) 6 weeks after alefacept treatment (Figure 3A and 3B). IL-20 expression in PsA lesional skin was positively correlated with disease activity (PASI score): *r *= 0.5062; *P *= 0.031 (Figure 3C).

## Discussion

Here we show that IL-20 is expressed in PsA synovium, similar to the levels in RA synovium. In PsA inflamed synovium, both CD68^+ ^macrophages and CD55^+ ^FLSs coexpress IL-20. This study is the first to investigate the effects of alefacept treatment on IL-20 expression in paired synovial tissue and psoriatic lesional skin of patients with both PsA and psoriasis. Whereas IL-20 expression in PsA synovium was not affected by alefacept treatment, its expression in lesional skin was significantly decreased after this treatment. In addition, in psoriatic lesional skin, IL-20 expression was positively correlated with disease activity (PASI) at baseline and after treatment. In contrast, synovial IL-20 expression was not correlated with disease-activity parameters (DAS28, CRP, ESR; data not shown). The observed expression of IL-20 in both PsA and RA synovial tissues is consistent with previous observations showing comparable levels of this proinflammatory cytokine in synovial fluid of patients with RA compared with those with spondyloarthritides, including PsA [24]. These data suggest that synovial IL-20 expression is a feature of various inflammatory rheumatic diseases.

As reported for RA [17,24], we found that both macrophages and FLSs are a source of IL-20 in the synovium of PsA patients. The exact biologic effects of synovial IL-20 have yet to be determined, but it is tempting to speculate that this cytokine might affect cells present in inflamed synovium. Consistent with this notion, it was reported that IL-20 induced the production of IL-6 and IL-8 and monocyte chemoattractant protein 1 by FLSs [17]**. **A considerable amount of evidence suggests that FLSs are key cells that contribute to RA pathogenesis. In this way, IL-20 could play a role in perpetuating the inflammatory process by promoting the release of inflammatory cytokines and chemokines at the site of inflammation [17].

Alefacept treatment resulted in changes in synovial inflammation in PsA patients that included a significant reduction in CD4^+ ^lymphocytes, CD8^+ ^lymphocytes, and CD68^+ ^sublining macrophages [4], supporting the hypothesis that T cells orchestrate synovial inflammation in PsA. However, the expression of synovial IL-20 was not affected by alefacept treatment, in contrast to the findings in the skin. This may be explained by the fact that alefacept treatment did not affect the numbers of intimal macrophages or the CD55^+ ^FLSs [4]; both cell types are sources of IL-20, but only the numbers of CD55^+ ^FLSs were positively correlated with the levels of synovial IL-20. Together, the data show that alefacept treatment does not affect the major cellular source of IL-20 in the synovium (FLSs), in contrast to the findings in the skin. Of note, alefacept treatment has been abandoned as a treatment of PsA, consistent with its limited clinical efficacy compared with TNF-α-targeted therapies [31-33]. As discussed, alefacept treatment was associated with decreased IL-20 expression in lesional psoriatic skin. As alefacept targets T cells, it is tempting to speculate that in the skin, T cells are involved in the proximal regulation of IL-20. It is well established that in psoriasis skin, cross-talk between infiltrating T cells and resident KCs is involved in disease pathogenesis [34]. In addition, it was recently reported that T cell-derived cytokines (IL-17 and IL-22) mediate IL-20 production by KCs [35]. Thus, in psoriatic skin of PsA patients, alefacept may affect KC-derived IL-20 by decreasing the number of infiltrating T cells that interact with KCs.

## Conclusions

IL-20 has been implicated in several inflammatory diseases. The results presented here show that IL-20 is expressed in psoriatic skin lesions and synovial tissue from patients with both psoriasis and PsA. It remains to be established whether the observed effects of alefacept are specific for IL-20 or also are seen with other members of the IL-10 subfamily (IL-19 and IL-24). Conceivably, the limited effectiveness of alefacept in PsA patients compared with anti-TNF therapy [31-33] might be explained in part by persistent IL-20 production by sublining macrophages and FLSs in the synovium.

## Abbreviations

ACR: American College of Rheumatology; AMC: Academic Medical Center; CRP: C-reactive protein; DAS28: disease activity score; DC: dendritic cell; ESR: erythrocyte sedimentation rate; FLS: fibroblast-like synoviocyte; HRP: horseradish peroxidase; ICH: International Conference of Harmonization; IL-20: interleukin-20; IL-20R: IL-20 receptor; IOD: integrated optical density; KC: keratinocytes; LFA-3: lymphocyte function-associated antigen-3; PASI: Psoriasis Area and Severity Index; PsA: psoriatic arthritis; RA: rheumatoid arthritis; SJC: swollen-joint count; TJC: tender-joint count; TNF: tumor necrosis factor; VAS: visual analogue scale.

## Competing interests

The authors declare that they have no competing interests.

## Authors' contributions

All authors meet the criteria for authorship and more specifically for contributorship statement: MCL and CLJ analyzed and interpreted the data and wrote the article under the close supervision of PPT and thereby take responsibility for this work. MCK, AWvK, JDB, MdR, DMG, and PPT conceived the study and participated in its design and coordination. MCK and AWvK collected and supplied the data. All authors agreed to publish this work and critically reviewed the article. Conception and design of this work were discussed with all authors.
